# Production of Mesoporous Magnetic Carbon Materials from Oily Sludge by Combining Thermal Activation and Post-Washing

**DOI:** 10.3390/ma15165794

**Published:** 2022-08-22

**Authors:** Wen-Tien Tsai, Yu-Quan Lin, Chi-Hung Tsai, Yun-Hwei Shen

**Affiliations:** 1 Graduate Institute of Bioresources, National Pingtung University of Science and Technology, Pingtung 912, Taiwan; 2 Department of Resources Engineering, National Cheng Kung University, Tainan 701, Taiwan

**Keywords:** oily sludge, pyrolysis-activation, mesoporous carbon composite, surface modification, pore analysis

## Abstract

In this work, the oily sludge (OS) from a local waste oil recycling plant was reused as a precursor for producing porous magnetic carbon composites (CC) by pyrolysis, followed by carbon dioxide activation. Based on the thermogravimetric analysis (TGA) of the OS feedstock, the preparation experiments were performed at 800–900 °C. From the pore analysis of the CC products, it indicated an increasing trend, as the BET surface area greatly increased from about 1.0 to 44.30 m^2^/g. In addition, the enhancement effect on the pore properties can be consistently obtained from the acid-washed CC products because the existing and new pores were reformed due to the leaching-out of inorganic minerals. It showed an increase from 32.27 to 94.45 m^2^/g and 44.30 to 94.52 m^2^/g at 850 and 900 °C, respectively, showing their mesoporous features. These porous and iron-containing features were also observed by the scanning electron microscopy-energy dispersive X-ray spectroscopy (SEM-EDS). In addition, the adsorption removal of total organic carbon (TOC) in the raw wastewater, by the CC product, showed its high performance (>80%).

## 1. Introduction

Industrial and transportation sectors that work with large numbers of machines and vehicles, using lubricating oil and engine oil, will need to store, transport, and treat large amounts of waste oil when repairing and during maintenance [1,2,3,4]. In addition, oily waste (or sludge) is regularly generated from the crude oil tank in the refinery industry [5]. Due to the residual oil remained, these collected waste oils will be off-site recycled to convert them into a generative resource. The recycled oil has a high calorific value, thus reusing it as an auxiliary fuel in certain sectors of industry is possible. However, a non-hazardous oily sludge will be generated from the physical separation process in the waste oil recycling plant. In Taiwan, this non-hazardous oily sludge (OS) has been coded as D-0903. Table 1 listed the reported amounts of the D-0903 waste for the past five years (2017–2021) in Taiwan by accessing the official database [6]. It indicated a decreasing trend in the reuse of this oily sludge as the treatment by self-combustion and off-site disposal increased accordingly. Obviously, these treatment approaches not only delete valuable resources but also increase operating costs.

OS is generally composed of petroleum-based oils, particulate matters, and metal oxides-containing rusts. In order to recycle the remaining hydrocarbons as economically as possible, many liquid–solid separation technologies have been adopted to recover residual oils from OS [5]. These methods included solvent extraction [7,8], distillation [9], mechanical centrifugation [10], surfactant [11], freezing/thawing [12], ultrasonic [12], and flotation [13]. Few studies focused on the thermochemical processes such as pyrolysis [14,15,16,17,18] and hydrothermal carbonization [19,20]. On the other hand, few studies focused on the production of carbon materials from OS by using activation processes [21,22,23,24,25,26,27,28,29]. However, the oily sludge samples used in these studies were taken from the generation sources, such as an oil refinery plant, not derived from the waste oil recycling plants. In addition, these studies adopted the chemical activation process for producing porous carbon materials from the oily sludge. Although the conversion of OS into porous carbons could be an available approach, the preparation method by chemical activation may not be an environmentally and economically promising one. As compared to the physical activation at higher activation temperatures, usually 800–900 °C [30], the chemical activation process can produce carbon materials, with higher carbon yields and larger pore properties, at lower activation temperatures (400–700 °C). However, the latter will generate wastewater containing chemical activating agents, such as zinc chloride (ZnCl_2_) and potassium hydroxide (KOH) [31].

It seemed feasible to recover the OS from the waste oil recycling plants because of the increasing costs and environmental risks of current waste treatment approaches. In addition, there are few published reports concerning the conversion of the OS into porous carbon composites (CC) using the carbon dioxide (CO_2_) physical activation. In the previous report [32], a preliminary test was performed at 850 °C as a function of residence time (30, 60, and 90 min), showing that the data on the Brunauer–Emmett–Teller (BET) surface area of the resulting products greatly decreased from 21.59 to 0.56 m^2^/g. In this work, we further performed the thermal activation experiments as a function of temperature (800–900 °C). To leach inorganic remains from the resulting CC and enhance their pore properties, we also treated them through dilute acid washing. Therefore, the pore and chemical properties of all CC products were determined by a nitrogen adsorption-desorption isotherm (−196 °C) and further observed by the scanning electron microscopy-energy dispersive X-ray spectroscopy (SEM-EDS).

## 2. Materials and Methods

### 2.1. Materials

The precursor for producing CC was a non-hazardous waste in the form of dark slurry (i.e., oily sludge, abbreviated as OS), which was obtained from a local waste oil recycling plant (Pingtung County, Taiwan). The as-received sample was immediately determined to get the thermochemical data on the thermogravimetric analysis, chemical analysis, and calorific value.

### 2.2. Thermochemical Properties of OS

In order to obtain the information about the conditions in the pyrolysis-activation experiments, the thermogravimetric (TG) instrument (TGA-51; Shimadzu Co., Kyoto, Japan) was used to determine the thermal behavior of the sludge, based on the weight loss percentage, as the temperature was programmed. As reported previously [33], the as-received sample (about 0.2 g) was placed into the TG instrument and heated from room temperature to 900 °C, at a fixed rate of 10 °C/min, with nitrogen gas flow of 50 cm^3^/min. In addition, the calorific value of the as-received OS sample was measured by an isoperibol oxygen bomb calorimeter (CALORIMETER ASSY 6200; Parr Co., Moline, IL, USA). Based on the isoperibolic mode at 25 °C, the calorific value analysis was performed in triplicate, using about 0.3 g of the sludge sample for each measurement. On the other hand, the elemental contents of the as-received OS sample were determined by the EDS. Although the EDS analysis is a rapid and simple means of chemical analysis, it can be combined with the beam-scanning functions of the scanning electron microscopy (SEM) to generate area maps for most elements detected [34]. In this work, the EDS instrument (7021-H; HORIBA Co., Kyoto, Japan) was used to detect the quantitative analysis (or distribution) of main elements (including carbon, oxygen, silicon, and other metals) on the surface of the dried OS sample.

### 2.3. Pyrolysis-Activation Experiments

In this work, the OS feedstock features its slurry semi-solid state containing high oil contents. Thus, the activation experiments for preparing a series of porous carbon composites from OS were based on the carbon dioxide (CO_2_) gasification, which were similar to those for preparing mesoporous carbon materials in a vertical furnace of inner diameter (ID) 10 cm and tube length 80 cm [35,36]. During the thermal process, we adopted a one-step model at a heating rate of about 10 °C/min. The experimental procedure involved the carbonization of dried OS (about 3 g for each experiment) below 500 °C, under the inert atmosphere, by flowing nitrogen gas (500 cm^3^/min) and then switched to the activation of the resulting char at elevated temperature (>500 °C), using CO_2_ (100 cm^3^/min) as a gasification gas. During the pyrolysis-activation experiments, the vaporized/vented gas was condensed by a cooling system for the production of fuel oil or pyrolytic oil, which has high heating value (ca. 42.0 MJ/kg).

As the activation reaction generally occurred at higher temperatures (i.e., 800–900 °C) [31], a series of preliminary experiments were performed by increasing the holding time, from nil to 90 min, at 800 °C. Based on the pore properties of the resulting carbon composites, the data on the BET surface area were in the range from 0.8 to 1.7 m^2^/g. Therefore, the subsequent experiments focused on the activation conditions at 850 and 900 °C without holding time, meaning that the electric furnace was powered-off when the temperature was reached. In order to enhance the pore properties of resulting CC products, we further used the dilute acid washing for removing inorganic components from them. Concerning the acid washing, the crude CC product was added to about 50 cm^3^ of 0.25 M HCl solution for thermal mixing on a hot-plate (about 75 °C for 30 min). After decanting the upper solution, the bottom slurry was rinsed with deionized water (100 cm^3^) three times to remove the residual inorganics. Finally, the acid-washed CC was dried in an air-circulating oven to perform the next procedure in the pore analysis. In this work, the resulting products were coded to compare with the data clearly. For example, OS-CC-850 and OS-CC-850-A referred to the CC products prepared from OS at 850 °C, without acid washing and with acid washing, respectively.

### 2.4. Characterization of OS-Based Carbon Composites (CC)

The pore analysis of the resulting CC products referred to the surface area, pore volume, and average pore size (diameter or width), which was based on the nitrogen (N_2_) adsorption-desorption isotherms, at −196 °C, in an accelerated surface area and porosimetry system (ASAP 2020; Micromeritics Co., Norcross, GA, USA). In order to obtain accurate data, it was required to remove all physically adsorbed materials from the sample surface before the N_2_ isotherm measurements. This was accomplished by vacuum pumping (≤1.33 Pa) at an elevated temperature (200 °C) to ensure a reproducible state of the sample surface. Using the corresponding model equations, such as Langmuir and BET [37,38], the data will indicate single-point surface area, Langmuir surface area, and BET surface area. Herein, single-point surface area was obtained from the single-point BET method, which reduced the experimental requirement to only one data point (at a relative pressure of about 0.3) under the assumption of a zero intercept (i.e., the value of C constant in the BET equation was taken as infinity.). In addition, the *t*-plot method was used to estimate the surface area and pore volume with micropores (pore diameter or width: <2.0 nm), based on the Harkins–Jura equation. Regarding the total pore volume, it is defined as the liquid (i.e., liquid nitrogen) volume at a certain saturated relative pressure (i.e., 0.95–1.00), where the adsorbed amount was converted into liquid volume by using liquid nitrogen density (i.e., 0.808 cm^3^/g at −196 °C). Furthermore, the average pore size was calculated from the values of the BET surface area and the total pore volume assuming that the pores with no surface (inner walls only) are of cylindrical geometry. On the other hand, the Barrett–Joyner–Halenda (BJH) method was used to calculate pore size distributions from experimental isotherms, using the Kelvin model of pore filling because it applies only to the mesopore (pore diameter or width: 2.0–50.0 nm) and small macropore (pore diameter or width: >50.0 nm) size range. In order to provide the textural structures of the resulting CC products, a scanning electron microscopy (SEM) (S-3000N; Hitachi Co., Tokyo, Japan), coupled with energy dispersive X-ray spectroscopy (EDS) (7021-H; HORIBA Co., Japan), was applied to observe the surface topography under an accelerating voltage of 15.0 kV. In order to provide the enhanced images with good contrast, a gold film using an ion sputter (E1010; Hitachi Co., Tokyo, Japan) coated the resulting CC samples in the SEM observations. In addition, an adsorptive test using the optimal CC was performed to evaluate the adsorption performance for the raw wastewater from the waste oil recycling plant. A raw wastewater containing a known amount of total organic carbon (TOC, about 11,000 mg/L) was prepared. About 100 mL of this solution was transferred into an Erlenmeyer flask with a defined amount of the carbon composite. Portions of the treated solution were filtered and then measured with the TOC analyzer (TORCH; Teledyne Tekmar Co., Mason, OH, USA).

## 3. Results and Discussion

### 3.1. Thermochemical Characterization of OS

The thermochemical characteristics of the feedstock greatly influence the design and performance of the thermal conversion system. Table 2 listed the data on the elemental (EDS) analysis and calorific value of the OS feedstock used in the thermal activation experiments. It showed that the measured data were different from the results of other OS feedstocks [21,22,23,24,25,26,27,28,29]. For example, the content of carbon in the OS was about 80.48 wt% in the study by Mohammadi and Mirghaffari [21]. In another study by Yang et al. [28], the content of carbon was as high as 84.38 wt%. In this work, the sludge obviously comprised a large carbon content (i.e., 61.46 wt%), causing a relatively higher calorific value (i.e., 24.34 MJ/kg). As expected, its carbon content was significantly lower than those in the published studies [21,28] where the oily sludge was collected from the fraction at the bottom of oil storage tanks in the petroleum refinery. On the other hand, the sulfur content (i.e., 3.51 wt%) in the OS feedstock were relatively high in comparison to those (0.7–1.41 wt%) of coal [39]. Consequently, the considerable emissions of sulfur oxides (SOx) may be released into the atmosphere when treating it by direct combustion in the waste incinerators and/or boilers.

The data on the contents of inorganic and mineral elements in the OS sample were also important because they will influence the design and operation of the thermochemical conversion system. Table 2 also listed the contents of relevant inorganic elements, including aluminum (Al), calcium (Ca), iron (Fe), and magnesium (Mg). Obviously, the content of Fe was significantly higher than other inorganic elements in the OS sample, which was consistent with those by Mohammadi and Mirghaffari [21]. The high contents of Fe or its oxides were likely connected with the rust contamination of the oil storage tank, thus creating a magnetic feature in the oily sludge sample.

**Figure 1:**
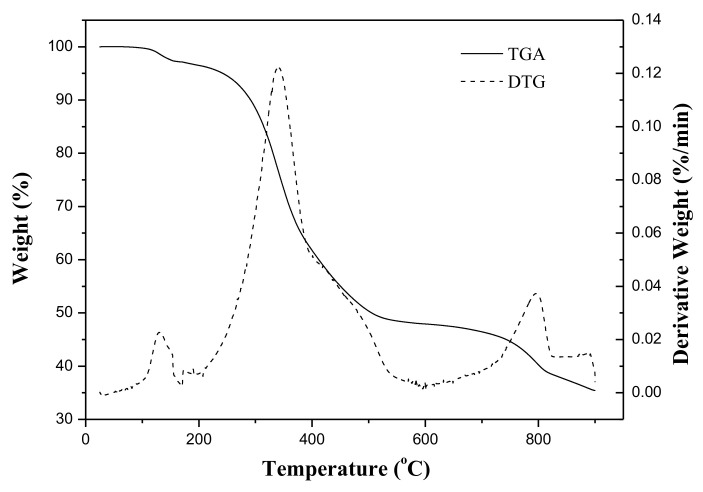
Thermogravimetric analysis/derivative thermogravimetry (TGA/DTG) curves of non-hazardous oily sludge (OS) at a heating rate of 10 °C/min.

The curves of thermogravimetric analysis (TGA) and its derivative thermogravimetry (DTG) for the as-received OS sample, at a heating rate of 10 °C/min under the nitrogen flow, were depicted in Figure 1, which was normalized based on the initial sample mass. Basically, the thermal behavior of the OS showed three main regions. A weight loss of about 5% was observed in the first stage, which can be ascribed to the evaporation of moisture and light organics from the sample during heating from 25 to 200 °C. The second weight loss was about 5–50%, which occurred between 200 and 500 °C. This mass loss should be related to the volatilization and pyrolysis decomposition of the organic fractions in the oily sludge. In this regard, the temperature of 500 °C was selected as the appropriate temperature for the preparation of the porous carbon (char) in the thermal activation experiments. The slight weight loss after 750 °C in the third stage was associated with the CO_2_ activation of resulting char and thermal decomposition of inorganic compounds such as calcium carbonate [21,40]. During the physical activation, the mass loss should be due to the gaseous product, carbon monoxide (CO), which evolved from the reaction between C (char) and CO_2_ [30,40]. Therefore, the activation temperature was set at 850 and 900 °C in this work, but the post-treatment experiments were performed to study the effects of acid-washing on the pore properties of resulting CC products.

### 3.2. Characterization of OS-Based CC

The specific surface area, pore volume, and pore diameter of the resulting CC products were listed in Table 3. Based on the BET surface area, it showed an increase from 32.27 m^2^/g (OS-CC-850) to 44.30 m^2^/g (OS-CC-900). This increment may be associated with enhanced gasification by CO_2_, thus creating more pores on the surface of CC at higher temperatures. On the other hand, the pore properties (using BET surface area for comparison) showed a consistent increase after acid-washing; i.e., 94.45 m^2^/g (OS-CC-850-A) and 94.52 m^2^/g (OS-CC-900-A). This enhancement effect can be elucidated by the dissolution of inorganic minerals, by means of acid-washing, in the previous studies [41,42], further leaching them out from the resulting CC products. Consequently, the existing and new pores in the resulting CC can be reformed and created, thus resulting in an increase in pore properties.

**Figure 2:**
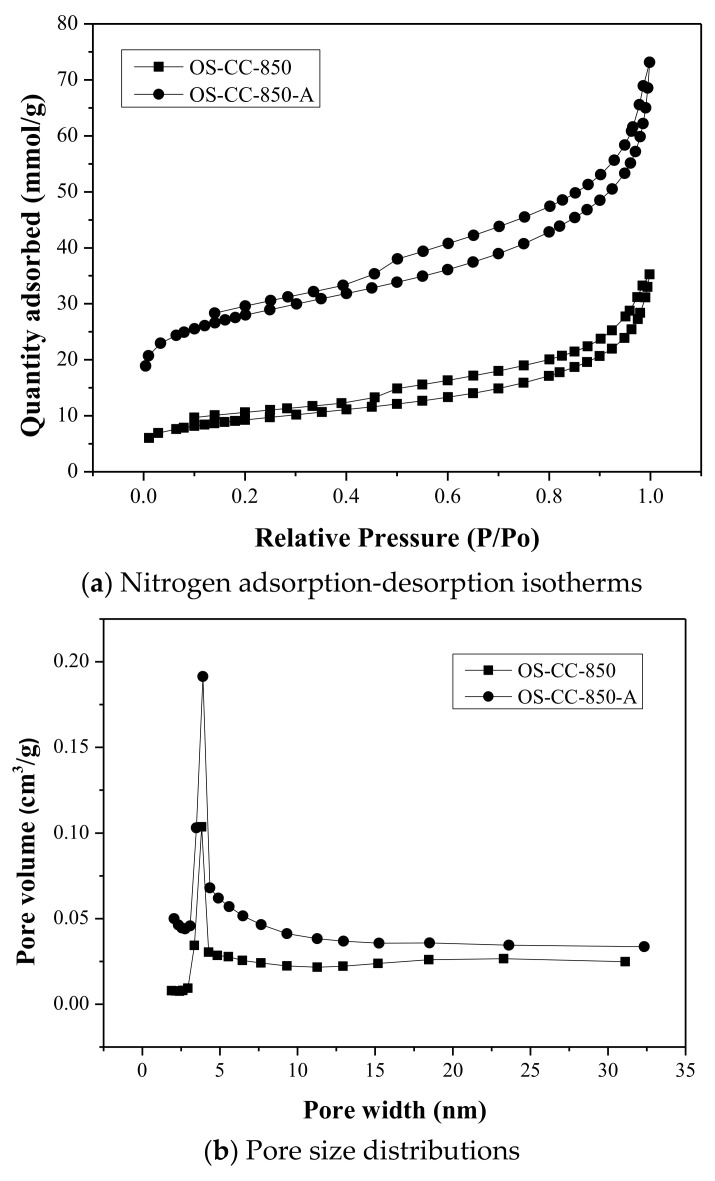
Nitrogen adsorption-desorption isotherms and BJH pore size distributions of CC products (i.e., OS-CC-850 and OS-CC-850-A).

**Figure 3:**
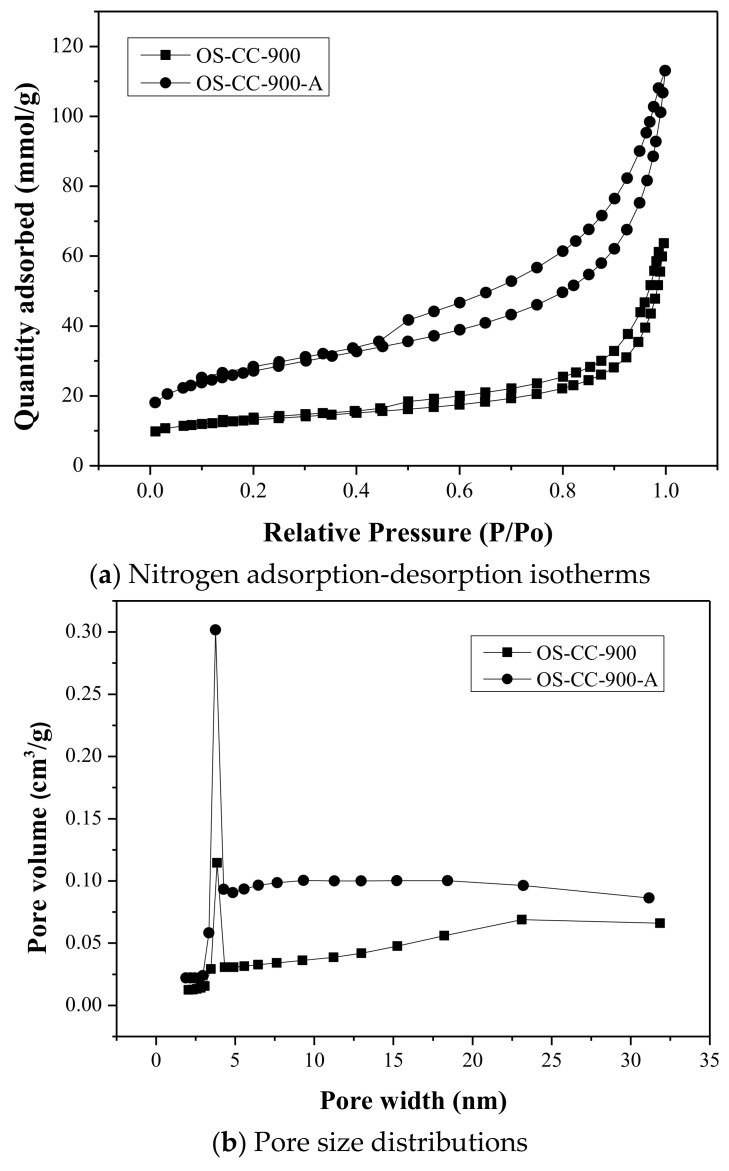
Nitrogen adsorption-desorption isotherms and BJH pore size distributions of CC products (i.e., OS-CC-900 and OS-CC-900-A).

The N_2_ adsorption/desorption isotherms and their corresponding pore size distributions for the resulting CC products (i.e., OS-CC-850 and OS-CC-900), as well as their acid-washed CC products (i.e., OS-CC-850-A and OS-CC-900-A), were shown in Figure 2 and Figure 3, respectively. According to the classification of adsorption isotherm by the International Union of Pure and Applied Chemistry (IUPAC) [38], the features of these plots belong to the type IV isotherms, which are typical for mesoporous materials, due to the hysteresis loop. This loop is associated with the occurrence of condensation in the mesopores. The initial adsorption stage of the type IV, over a range of low relative pressure, can be attributed to monolayer-multilayer adsorption, such as the type II isotherms, which are typically obtained in the case of nonporous or macroporous materials. Furthermore, according to the IUPAC classification of hysteresis loops, the type H3 hysteresis was observed in the resulting CC products, indicating that the plate-like particle aggregates (e.g., certain clays), or macropores, were in the CC. The desorption branch for type H3 hysteresis also contained a steep region, which occurred, for nitrogen, at −196 °C in the relative pressure from 0.4. Depicted in the right side of Figure 2 and Figure 3, it was also consistent with the data on the pore properties (Table 3) because the isotherm curves of the acid-washed CC (i.e., OS-CC-850-A) were positioned on the upper side. Obviously, these CC products (i.e., OS-CC-850-A and OS-CC-900-A) are porous materials by featuring their mesopores and macropores. Based on the BJH model, the left side of Figure 2 and Figure 3 showed the plots of pore size distribution for the resulting CC products (i.e., OS-CC-850/OS-CC-850-A and OS-CC-900/OS-CC-900-A), indicating a mesopore size distribution in the narrow range of 3.0 to 4.5 nm.

**Figure 4:**
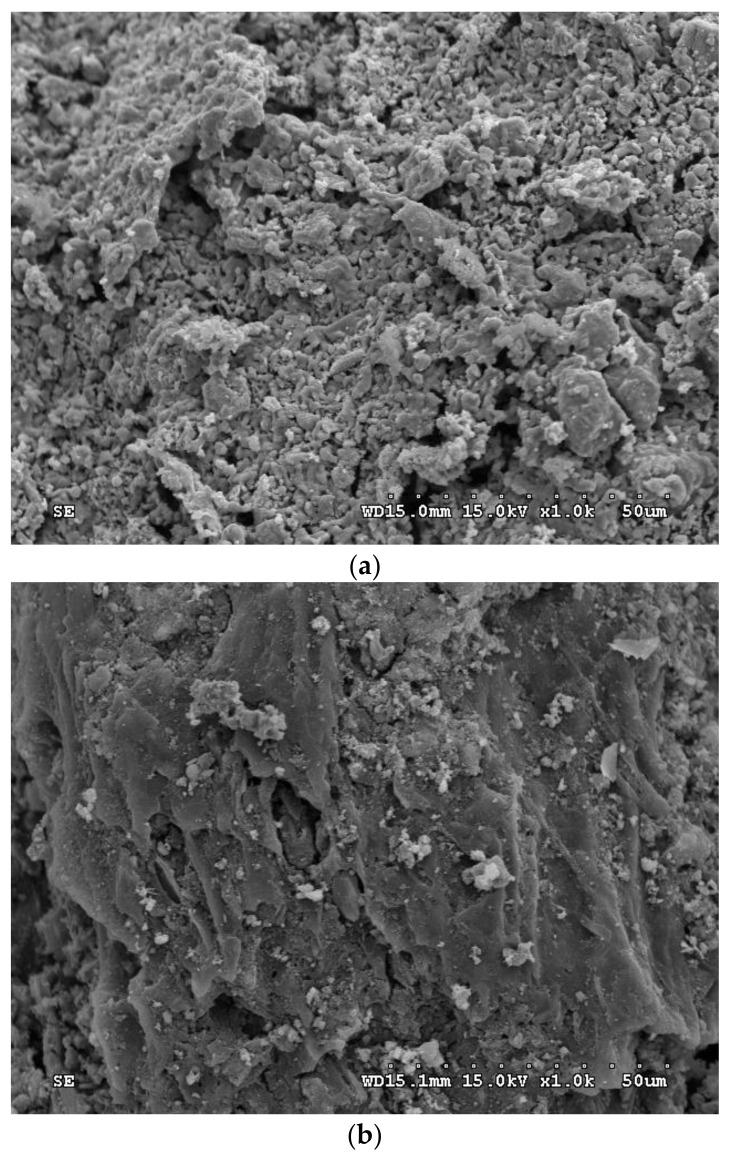
SEM images (×1000) of the optimal CC products for (**a**) OS-CC-900 and (**b**) OS-CC-900-A.

**Figure 5:**
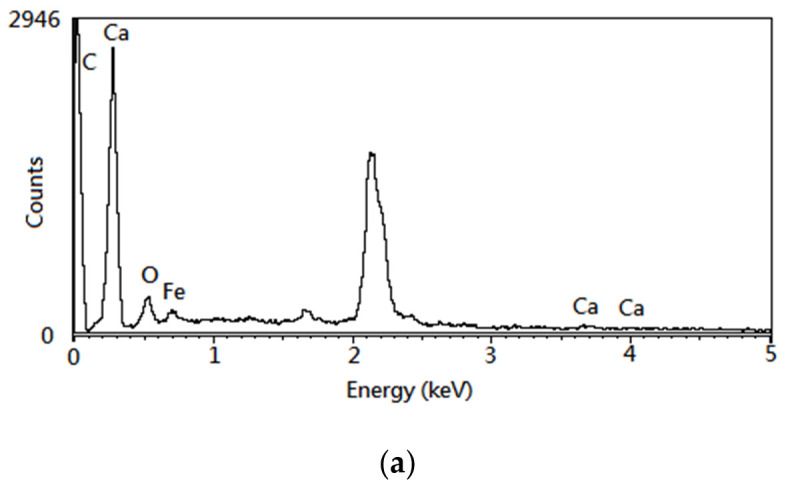

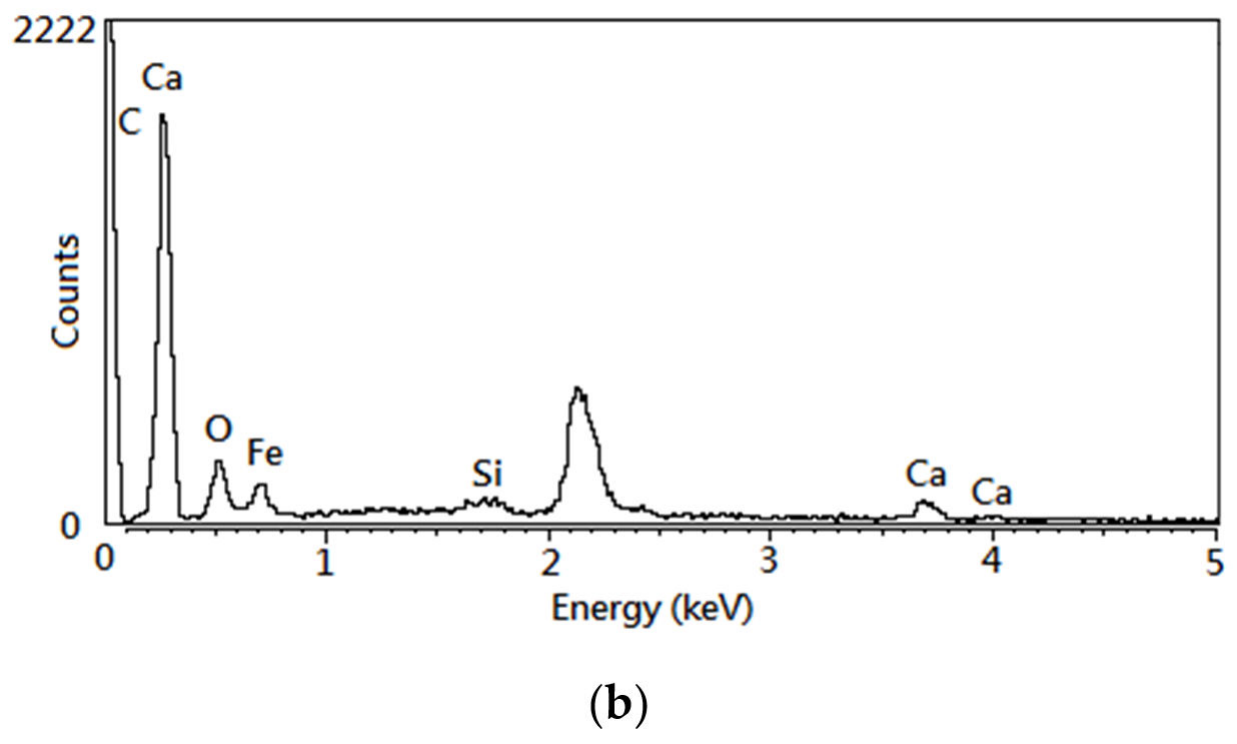
EDS spectra of the acid-treated CC products for (**a**) OS-CC-850-A and (**b**) OS-CC-900-A.

The SEM image of the optimal CC product (i.e., OS-CC-900) showed a pitted and heterogeneous surface (Figure 4a), having different mesopore/macropore-scale holes. The presence of many grains observed on the surface could be related to the structures of inorganic minerals such as iron oxides (Fe_2_O_3_/Fe_3_O_4_) and aluminum oxide (Al_2_O_3_). By contrast, the acid-washed CC (i.e., OS-CC-900-A) indicated a clean surface with more pores (Figure 4b), which were reformed and created during the acid-leaching. The SEM observations were in accordance with the data on their pore properties in Table 3. Based on the elemental analysis on the surface of CC products (i.e., OS-CC-850-A and OS-CC-900-A) by the EDS (Figure 5), the contents of C/O/Ca/Fe are 69.23/15.03/0.67/15.07 wt% and 59.12/14.29/2.17/23.99 wt%, respectively. Obviously, the high contents of iron (Fe), oxygen (O) and carbon (C) in the resulting CC products were indicative of its features in oxygen-containing functional groups and magnetic iron oxides on the surface. In this regard, the resulting mesoporous Fe/CC could be an effective adsorbent, which can be easily separated from aqueous solutions by applying external magnets for reusing it [43,44].

After the adsorption tests by using different adsorbent dosages with the optimal CC product (i.e., OS-CC-900-A, BET surface area ≈ 100 m^2^/g), the concentrations of total organic carbon (TOC) in the raw wastewater can be reduced from about 11,000 to 2000–9500 mg/L, giving the highest removal efficiency by 81.8% TOC.

## 4. Conclusions

In this work, the oily sludge (OS) from a local waste oil recycling plant was reused as a precursor for producing porous carbon composites (CC) by thermal activation processes. Based on the results by the thermogravimetric analysis (TGA) of the OS feedstock, the preparation experiments were performed at 800–900 °C. From the pore a sharp increase in the BET surface area between 800 and 850 °C. This decline may be associated with enhanced gasification by CO_2_ and the calcination of minerals (i.e., calcium carbonate), thus causing the removal of limited carbon on the surface of activated char at higher temperatures. In addition, the enhancement effect on the pore properties can be consistently obtained from the acid-washed CC products because the existing and new pores were reformed because of the leaching-out of inorganic minerals. It showed significant increases from 32.27 to 94.45 m^2^/g at 850 °C and 44.30 to 94.52 m^2^/g at 900 °C. It was obvious that the mesopores and macropores were concurrently formed in the resulting CC products based on the N_2_ adsorption/desorption isotherms. In this regard, the resulting CC could be an effective adsorbent for removal of large-molecule adsorbates from aqueous solution. On the other hand, further research is necessary to optimize the activation processes for producing CC with higher pore properties.

## Figures and Tables

**Table 1:** Reported amounts of non-hazardous oily sludge (D-0903) treatment in Taiwan [6].

Year	Reuse	Self-Treatment	Commissioned or Joint Treatment	Total
2017	1744	4627	10,519	16,890
2018	887	7113	10,019	18,019
2019	636	10,572	8758	19,966
2020	490	10,776	10,858	22,124
2021	907	7951	10,802	19,660

**Table 2:** EDS analysis and calorific value of oily sludge.

Property	Value
EDS analysis ^a,b^	
Carbon (wt%)	61.456
Oxygen (wt%)	15.952
Sulfur (wt%)	3.508
Aluminum (wt%)	3.508
Calcium (wt%)	6.457
Iron (wt%)	11.352
Magnesium (wt%)	0.938
Calorific value (MJ/kg) ^a,^^b^	24.34 ± 0.31

^a^ Air-dry basis (as received sample). ^b^ The mean ± standard deviation for three determinations.

**Table 3:** Pore properties of resulting carbon composites.

Pore Property	OS-CC-850	OS-CC-850-A	OS-CC-900	OS-CC-900-A
Specific surface area (m^2^/g)				
Single point surface area ^a^	31.04	94.62	44.41	91.06
BET surface area ^b^	32.27	94.45	44.30	94.52
*t*-plot micropore area ^c^	7.62	40.59	18.51	21.30
*t*-plot external surface area	24.65	53.85	25.79	73.22
Pore volume (cm^3^/g)				
Total pore volume ^d^	0.052	0.106	0.093	0.166
*t*-plot micropore area ^c^	0.004	0.020	0.009	0.010
Pore size (nm)				
Average pore width ^e^	6.40	4.49	8.36	7.04

^a^ By the single point BET method at relative pressure of 0.30. ^b^ By the BET method at relative pressure range of 0.06–0.30 (9 points). ^c^ by the *t*-plot method. ^d^ By the single point adsorption at relative pressure of 0.995. ^e^ By the ratio of the total pore volume (V_t_) to the BET surface area (S_BET_) (i.e., Average pore width = 4 × V_t_/S_BET_).

## Data Availability

Not applicable.
